# Biases and blind spots in the global research agenda on metallic pollution and bees

**DOI:** 10.1007/s00114-026-02125-z

**Published:** 2026-06-08

**Authors:** Fernando M. T. Martins, Cristiano Schetini de Azevedo, Diego G. Rocha, Diego H. A. de Melo, Marcelo C. Ribeiro, Sandra A. L. de Moura

**Affiliations:** 1 Department of Environmental Engineering, School of Mines, Federal University of Ouro Preto, Campus Morro do Cruzeiro, Street Quatro, Bauxita, CEP: 35.402-136, Ouro Preto, Minas Gerais Brazil; 2 Department of Biodiversity, Evolution, and Environment, Institute of Exact and Biological Sciences, Federal University of Ouro Preto, Campus Morro do Cruzeiro, Street Quatro, Bauxita, CEP: 35.402-136, Ouro Preto, Minas Gerais Brazil; 3 Department of Chemistry, Federal University of Minas Gerais, Belo Horizonte, Minas Gerais 31270-901 Brazil; 4 Department of Statistics, Institute of Exact and Biological Sciences, Federal University of Ouro Preto, Campus Morro do Cruzeiro, Street Quatro, Bauxita, CEP: 35.405-136, Ouro Preto, Minas Gerais Brazil

**Keywords:** Metallic pollutants, Toxicity, Bees, Environmental impacts, Scientometric analysis

## Abstract

Metallic pollution is an emerging and underappreciated stressor contributing to global bee declines, yet the evidence base remains fragmented across metals, taxa, endpoints, and geographic regions. Here, we provide the first hypothesis-driven scientometric synthesis of the global literature on metallic pollutants and bees. Using Web of Science records, we compiled 154 experimental and field-based studies and quantitatively tested five predictions regarding taxonomic, methodological, and thematic biases. Publication output increased sharply after 2013, with China, the United States, and Brazil leading research activity. Research effort was disproportionately concentrated on managed honey bees (*Apis mellifera* and *Apis cerana*), adult workers, and dietary exposure pathways, while larvae, wild bee taxa, and environmentally mediated exposures were less examined. Physiological, behavioural and mortality endpoints dominated the literature, whereas reproductive and microbiome impacts remained major blind spots. Adverse outcomes predominate across metals, although essential elements (Zn, Cu, Se) tended to produce less severe effects than non-essential toxic metals (Cd, Pb, Hg), this difference disappeared when exposure concentration and duration were considered. Experimental concentrations varied significantly among metals in food-based exposure studies, indicating substantial heterogeneity in dosing regimes across the literature. Multi-metal studies were not statistically underrepresented, although only a narrow subset of metal combinations has been repeatedly tested. Overall, this synthesis highlights strong structural biases in current knowledge production and identifies priorities for future research, including broader taxonomic coverage, inclusion of early life stages, standardised field-realistic exposures, and integration of chronic, sublethal, and multi-stressor scenarios. Expanding evidence toward more ecologically representative designs will be essential for robust risk assessment of metallic contamination in pollinator communities.

**Supplementary Information:**

The online version contains supplementary material available at 10.1007/s00114-026-02125-z.

## Introduction

Bees play a fundamental role in maintaining ecosystem functioning through pollination (Kevan and Viana 2003). Despite their ecological and economic importance, bee populations have declined worldwide in recent decades (Hallmann et al. 2017). During foraging, bees are exposed to a wide range of environmental stressors, including pathogens, pesticides, habitat degradation, and metal pollutants (Cunningham et al. 2022). These elements can be ingested or transported to colonies through nectar, pollen, water, and nesting materials (Bargańska et al. 2016). Because bees integrate contaminants from multiple environmental compartments into their bodies and products, they are widely considered valuable bioindicators of environmental pollution, particularly metallic contamination (Farias et al. 2023; Hussein et al. 2024; Joshi and Deokar 2024).

Metallic pollutants typically occur in environmental matrices as complex mixtures rather than isolated elements (Monchanin et al. 2023). Interest in their ecological impacts has increased in recent years, as studies indicate that even concentrations below human safety thresholds may produce harmful effects on pollinators (Monchanin et al. 2021b). Nevertheless, the current evidence base remains fragmented and unevenly distributed across metals, bee taxa, biological endpoints, and geographic regions (Hladun et al. 2015a; Bargańska et al. 2016; Naccari et al. 2025).

Research in bee ecotoxicology has historically focused on a small number of model organisms, particularly the managed honey bee *Apis mellifera*, due to its economic importance and ease of laboratory handling (Arena and Sgolastra 2014; Sanchez-Bayo and Goka 2014). This emphasis has been reinforced by regulatory frameworks that use honey bees as the primary surrogate species for assessing pollinator risks in ecotoxicological testing (European Food Safety Authority 2013; USEPA - United States Environmental Protection Agency 2014). For instance, honey bees are generally reliable surrogates for LD₅₀-based pesticide risk assessments (Thompson and Pamminger 2019). However, bee taxa differ widely in life history traits, nesting biology, physiology, and exposure pathways (Sgolastra et al. 2019; Franklin and Raine 2019). Consequently, reliance on a restricted set of model species may limit the ecological generalisation of toxicological findings. Furthermore, experimental designs often focus on adult individuals and dietary exposure routes, typically via contaminated pollen or nectar, while other environmentally relevant pathways, such as contact with contaminated soil, dust, or nesting substrates, remain comparatively understudied. We therefore hypothesise (H1) that research effort is disproportionately concentrated on *A. mellifera* and a few additional model species, with adult bees and dietary exposure pathways strongly overrepresented in the literature.

Another limitation concerns the biological responses that are most frequently evaluated. Many toxicological studies prioritise readily measurable outcomes such as mortality and physiological impairment, whereas more complex but ecologically relevant endpoints, including reproduction, colony development, behavioural disruption, and microbiome-mediated effects, remain comparatively rare (Gekière et al. 2023; Li et al. 2024; Esmaeily et al. 2025). Such imbalances may hinder the detection of subtle but potentially important population-level consequences of metal exposure. Accordingly, we hypothesise (H2) that lethal and physiological endpoints predominate in published studies, whereas colony-level, reproductive, behavioural, and microbiome-related impacts remain underrepresented.

Metals also differ fundamentally in their biological roles. While elements such as Zn, Cu, and Se are essential micronutrients involved in enzymatic activity and antioxidant defence, others, including Cd, Pb, and Hg, have no known physiological function and are primarily toxic (Rengel 2004; Tchounwou et al. 2012; Monchanin et al. 2021c; Naccari et al. 2025). This distinction raises the possibility that reported outcomes may differ systematically between essential and non-essential metals. However, toxicity ultimately depends on exposure dose and duration, and even essential elements may produce adverse effects when physiological thresholds are exceeded. We therefore hypothesise (H3) that essential metals are more frequently associated with neutral or positive outcomes compared with non-essential metals.

Experimental conditions also vary widely across studies. Administered concentrations often differ by several orders of magnitude, complicating cross-study comparisons and limiting the ecological realism of laboratory exposure scenarios (Chapman 2002; Martin et al. 2021). Because environmental concentrations of metals in bee-relevant matrices such as pollen, nectar, dust, and water remain poorly characterised, it is often difficult to determine whether experimental exposures reflect realistic field conditions. We therefore expect (H4) that administered concentrations vary substantially both among and within metals across studies.

Finally, although metallic pollutants frequently occur as mixtures in natural environments, experimental research often evaluates individual metals in isolation. Considering the likelihood of simultaneous exposure to multiple contaminants, we hypothesise (H5) that studies assessing multi-metal mixtures remain relatively rare.

To address these questions, we conducted a quantitative synthesis of the global literature on metallic pollutants and bees using scientometric approaches (Schubert 2001; Parra et al. 2019). A recent synthesis has provided a comprehensive mechanistic overview of how trace metals affect bees, highlighting exposure routes, detoxification processes, and impacts across multiple biological levels (Gekière et al. 2023). While this work represents an important step toward understanding the physiological and behavioural mechanisms underlying metal toxicity in bees, it was not designed to systematically evaluate how research effort is distributed across species, life stages, metals, exposure pathways, and impact categories. Building on this foundation, our study adopts a complementary quantitative perspective to identify structural patterns in the production of knowledge in this research field.

Specifically, we compiled a global dataset substantially larger than that used in the previously mentioned study and analysed patterns in publication trends, geographic distribution, metals investigated, bee taxa studied, life stages considered, exposure pathways, and biological endpoints. This approach allows us to identify research biases, recurring experimental designs, and major knowledge gaps that may influence current interpretations of the ecological risks posed by metallic pollution to bees.

In addition, we introduce a Net Effect framework to summarise the predominant direction of reported outcomes across studies. Based on a vote-counting logic, this approach classifies reported responses as predominantly negative, neutral, or positive within each metal–impact category (Bushman and Wang 2009; Cumpston et al. 2023). Accordingly, Net Effect scoring should be interpreted as an exploratory indicator of the predominant direction of reported responses rather than as a quantitative estimate of effect magnitude. Although it does not estimate effect sizes as formal meta-analyses do, this method provides a useful synthesis of highly heterogeneous studies, highlighting metals consistently associated with adverse responses and identifying underexplored biological endpoints (Borenstein et al. 2009; Haddaway et al. 2016). Directional synthesis approaches of this type have been widely used to summarise patterns of evidence and identify consistent trends across complex datasets (Hedges and Olkin 1985; Bushman and Wang 2009; Koricheva et al. 2013; Higgins et al. 2019). By combining scientometric analysis with Net Effect scoring, our study aims to provide a structured overview of current research patterns and identify metals and biological endpoints that remain underexplored. Such insights may help improve ecological risk assessment and guide future research on metallic contamination in pollinator communities.

## Materials and methods

### Database selection and search criteria

The database chosen for the literature searches and selection was the Web of Science (WoS). This is one of the most widely used databases for bibliometric and scientometric analyses worldwide, encompassing a diverse range of works on multidisciplinary topics (Singh et al. 2021; Lim et al. 2024).

Paper searches followed the recommendations of the Preferred Reporting Items for Systematic reviews and Meta-Analyses (PRISMA) Protocol (Page et al. 2022). PRISMA was adopted here not to conduct an effect-size meta-analysis, but to ensure transparency and reproducibility in constructing a bibliometric evidence base for hypothesis-driven mapping. The keywords chosen to compose the database search are presented below and were divided into three parts, connected by the Boolean operator “AND”:

- First part, referring to bees: (bee OR bees OR honeybee* OR bumblebee* OR apis)

AND

- Second part, referring to metallic, metalloid, or trace elements: (metal OR metals OR metallic OR trace OR metalloid OR metalloids)

AND

- Third part, to delimit works related to impacts (environmental or bee-related), or pollutants or pollution, or the environment, or health (animal or environmental), or analyses (chemical, biological, biochemical), or monitoring or monitors, or biomonitoring or biological monitors, or toxicity, or toxic, or intoxication or intoxicating, or biological markers: (Impact* OR pollut* OR environment* OR health OR analysis OR biomonitor* OR monitor* OR toxic* OR intoxic* OR biomarker).

The asterisk is used in WoS as a wildcard character, representing any character or group of characters, including no character, and serving to replace these possible characters in a word (Clarivate 2022). It is worth noting that for the first part of the keywords defined for the article search, all bee genera found in (Michener 2007) were tested; however, only *Apis*, in addition to the bumblebee, showed an increase in search results. Therefore, these two words remained.

**Fig. 1:**
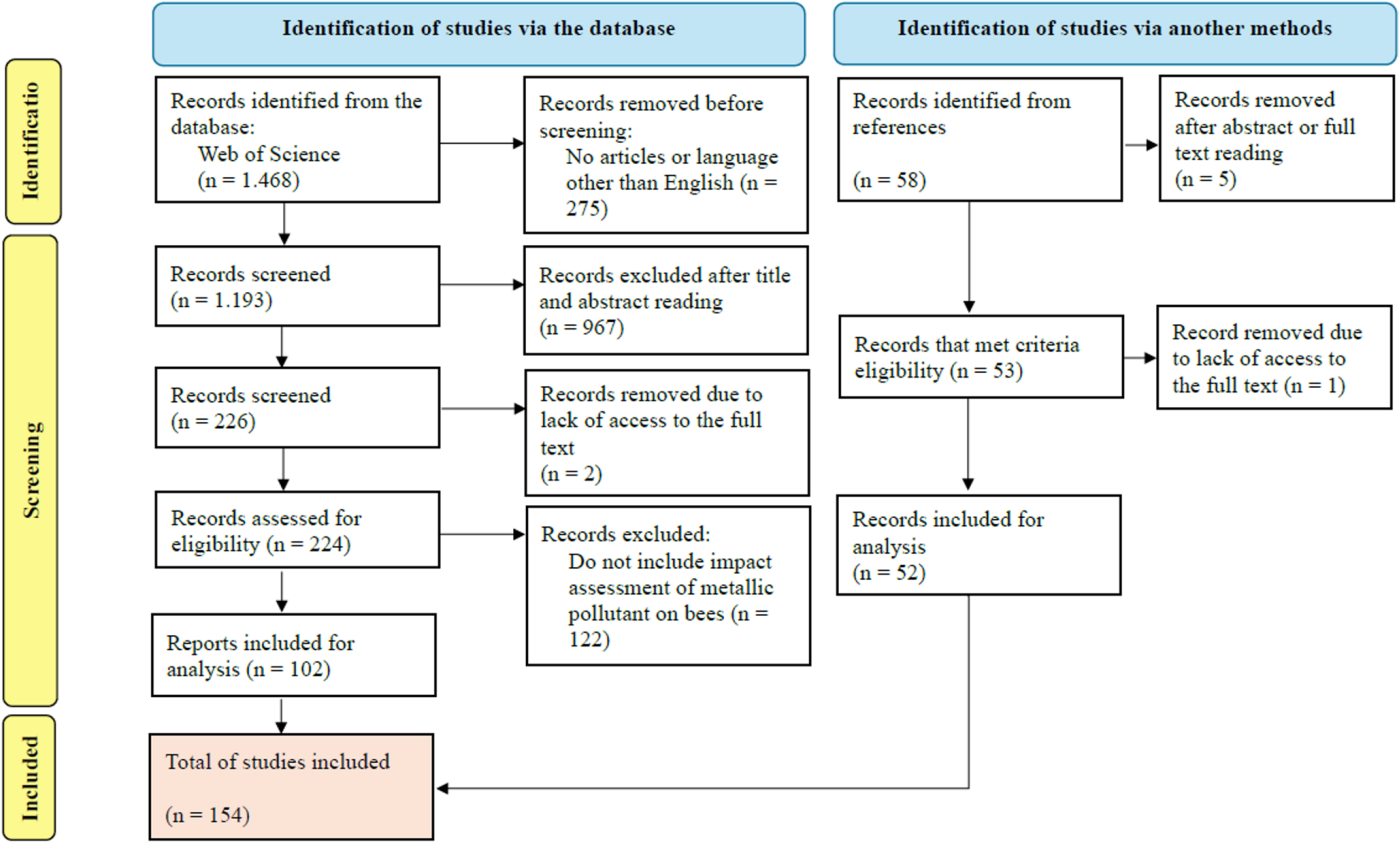
PRISMA flow diagram summarising the stages of selecting scientific literature for the scientometric study. The illustration presents the counts of records identified, assessed, and incorporated based on the specified inclusion and exclusion parameters

The documents were searched across the entire period available on the platform up to December 31, 2025. For all articles resulting from the bibliometric searches, the following inclusion criteria was adopted: (1) the study must be original, include fieldwork (sampling) or laboratory work; (2) the study must analyse bees; (3) the study must deal with metallic pollutant (or pollutants); and (4) the study must present the results obtained from the interaction between bees and metallic pollutants, represented by reported directional outcomes (negative, neutral/absent, or positive), which were later synthesized through Net Effect scoring. For studies conducted at the community level (e.g., surveys reporting responses of multiple bee species), each species reported in the study was treated as an individual entry in the dataset to allow species-level analyses. However, all entries remained linked to the same source publication to avoid inflating the total number of independent studies. More than 1,400 papers were retrieved through the Web of Science search, and additional records were identified via reference list screening (snowballing). After title/abstract screening and full-text evaluation, 154 studies remained for the final analysis (Fig. 1).

The considered impacts were divided into eight classes: (1) cytological and histological, (2) physiological, (3) on the gut microbiota, (4) phenotypic, (5) behavioural, (6) reproductive, (7) mortality, and (8) colony, based on an established ecotoxicological categorization scheme proposed by (Gekière et al. 2023), which we adopted to ensure comparability across studies. Importantly, whereas (Gekière et al. 2023) provided a mechanistic narrative synthesis, we use these categories as input variables for quantitative scientometric hypothesis testing and gap detection. For statistical analyses based on frequency distributions (e.g., Chi-square tests), impact categories represented in fewer than five articles were pooled into a single “Other” group to reduce sparsity and improve statistical robustness. However, for the directional Net Effect synthesis, impact classes were retained individually to preserve the ecotoxicological interpretation of specific biological endpoints. This approach allowed us to retain the full breadth of impacts reported across the literature while avoiding unstable frequency estimates driven by extremely low counts. Studies involving radionuclides were excluded, even if they also involve metallic elements, because they focus on radioactive unstable isotopes (ATSDR 2008), which are not the subject of this research.

### Scientometrics and statistical analysis

Scientometric data are presented in both absolute and relative terms, as appropriate, since some studies assessed more than one bee species, multiple metallic pollutants or concentrations, and several impact categories. Consequently, summed counts may exceed the total number of articles included in the analyses. All analyses were conducted in R (version 4.5.2) (R Core Team 2025), adopting a significance level of 5% (*p* < 0.05).

First, in an exploratory analysis, a piecewise (broken-stick) linear regression was used to assess whether publication trends differed between earlier and later phases of the time series. This analysis was intended to characterise the temporal dynamics of publication growth rather than to provide a predictive model of future publication output. Annual publication counts were modelled as a continuous function of year, allowing the slope to change after a reference transition point. Based on visual inspection of the publication trajectory, the year 2013 was adopted as a pragmatic breakpoint separating an initial period of low output from a subsequent phase of sustained growth (Toms and Lesperance 2003; Muggeo 2003).

The model was fitted as:

$$\:y={\beta\:}_{0}+{\beta\:}_{1}\left(\mathrm{year}\right)+{\beta\:}_{2}(\mathrm{year}-c{)}_{+}$$

where * y* is the number of publications per year, * c* is the reference year (2013), and $\:\left(\mathrm{year}-c)_+\right.$ equals zero before * c* and increases linearly thereafter. In this formulation, $\:{\beta\:}_{1}$represents the slope before the transition year, whereas $\:{\beta\:}_{1}+{\beta\:}_{2}$ represents the slope after 2013.

Additionally, country information was retrieved from Web of Science metadata based on the research location, rather than on the first author’s affiliation. A heatmap showing the number of studies conducted per country was constructed using the ggplot2 (Wickham 2016) and ggspatial (Dunnington 2017) packages. Additional heatmaps were generated to examine the relationships among metallic pollutants, bee species, and study locations, thereby synthesising the geographic and taxonomic distribution of research efforts.

To test Hypothesis 1 (H1), regarding disproportionate research attention among bee species, life stages (adults, larvae, or both), castes (drone, queen, worker, solitary, or more than one), metals, and exposure media, Chi-square goodness-of-fit tests were applied to publication counts. Exposure media reported in the reviewed studies were consolidated into broader categories to allow a more interpretable assessment of methodological bias. Specifically, the original exposure media were grouped into three main classes: Food (e.g., syrup, pollen, larval food, and water-based administration, corresponding primarily to oral exposure), Environment (e.g., soil, wax, plantations, irrigation water, or other contaminated substrates where bees are exposed through environmental contact or interaction with the surrounding matrix), and Body (e.g., direct application to the organism, including topical contact treatments and injection-based approaches).

To test Hypothesis 2 (H2), regarding the predominance of lethal and physiological endpoints in published studies and the underrepresentation of colony-level, reproductive, and microbiome impacts, a Correspondence Analysis (CA) was applied as an exploratory ordination method to visualise associations between metallic pollutants and impact classes based on patterns of co-occurrence in the literature (Greenacre 2017). CA was performed on a contingency table summarising the frequency of treatment-level records for each metal–impact class combination, capturing the thematic and intellectual structure of research efforts rather than quantitative measures of effect magnitude. Analyses were conducted using the FactoMineR package (Lê et al. 2008). In addition, a Chi-square goodness-of-fit test was used to assess whether the number of studies differed significantly across impact classes.

To complement this frequency-based mapping, Net Effect scores were calculated as directional indicators of reported outcomes, coding each treatment-level record as positive, negative, or neutral. These scores were used to visualise whether the most frequently studied metal–impact combinations were predominantly adverse, positive, or neutral.

For H2 and H3 analyses, directional Net Effect indices were calculated for each metal–impact combination to synthesise the overall direction of reported outcomes. This approach provides a form of directional evidence synthesis conceptually related to vote-counting methods used in meta-analysis when standardised effect sizes cannot be consistently extracted across heterogeneous experimental designs (Hedges and Olkin 1985; Bushman and Wang 2009).

For each treatment-level record, outcomes were coded according to their reported direction: positive (+ 1), negative (− 1), or neutral/no effect (0). To allow comparison across metal–impact combinations with different numbers of observations, Net Effect scores were normalised by the total number of available records. The resulting index therefore represents the mean directional outcome across studies and ranges from − 1 (all effects negative) to + 1 (all effects positive):

$$\:N{E}_{\left(m,i\right)}=\frac{1}{n}\sum\:_{k=1}^{n}{s}_{k}$$

where $\:{s}_{k}$ represents the directional score of the * k*-th treatment-level observation and * n*is the total number of records available for that metal–impact combination.

Because biological responses to contaminants depend not only on exposure but also on its magnitude and duration, we additionally performed a weighted directional synthesis incorporating both administered concentration and exposure time. This weighting scheme reflects the well-established dose–time relationship in ecotoxicology, whereby toxicological responses depend on both exposure intensity and duration (National Research Council (US) 2011; Newman and Newman 2014; Walker et al. 2016).

Weighted Net Effect scores were therefore calculated by multiplying each directional score by a weight representing treatment intensity:

$$\:WN{E}_{\left(m,i\right)}=\frac{\sum\:_{k=1}^{n}{s}_{k}\cdot\:{w}_{k}}{\sum\:_{k=1}^{n}{w}_{k}}$$

where the weight $\:{w}_{k}$ was defined as:

$$\:{w}_{k}=\mathrm{l}\mathrm{o}\mathrm{g}(1+{C}_{k})\times\:\mathrm{l}\mathrm{o}\mathrm{g}(1+{T}_{k})$$

with $\:{C}_{k}$ representing the administered concentration (mg L⁻¹) and $\:{T}_{k}$the exposure duration (days). Logarithmic transformation was applied to both variables to reduce the influence of extreme values and stabilise variance across studies with widely differing exposure regimes. This transformation also reflects common practice in ecological and toxicological modelling when integrating exposure metrics spanning multiple orders of magnitude (Newman and Newman 2014; Walker et al. 2016). The resulting weighted index ranges from − 1 to + 1 and reflects the balance of reported outcomes, giving proportionally greater weight to treatments with higher concentrations and longer exposure durations.

To test Hypothesis 3 (H3), regarding whether essential metals (e.g., Zn, Se, Cu) are more frequently associated with neutral or positive outcomes compared with non-essential toxic metals (e.g., Cd, Pb, Hg), metals were grouped according to their biological role. Differences between these groups were evaluated using a Wilcoxon rank-sum test applied to both unweighted Net Effect indices and the weighted Net Effect metric across metal–impact combinations. This non-parametric approach was chosen because Net Effect values represent directional summary indices rather than normally distributed effect sizes, and because the number of available observations differed among metals, making distribution-free methods more appropriate for group comparisons.

Importantly, the database was structured at the treatment level rather than at the article level. Thus, when a single study investigated multiple metals, concentrations, exposure durations, bee species, or experimental comparisons, each distinct metal–dose–exposure-time combination was recorded as a separate treatment-level entry in the dataset and contributed individually to the CA frequency table and Net Effect scoring. This design prevents the conflation of multiple treatments into a single directional outcome and ensures that concentration-dependent and duration-dependent contrasts are represented in the synthesis. Because the objective of this analysis was to summarise the directional balance of reported outcomes rather than to estimate standardised effect sizes, Net Effect scores were used primarily as qualitative directional indicators rather than as part of a formal quantitative meta-analysis. Accordingly, treatment-level entries represent distinct experimental comparisons within studies rather than statistically independent replicates. This approach enabled us to summarise whether published evidence for a given metal or metal–impact combination was predominantly adverse, positive, or neutral.

To test Hypothesis 4 (H4), regarding whether administered experimental concentrations differed significantly among metals, the Kruskal–Wallis test was applied, followed by Dunn’s post-hoc comparisons with Bonferroni correction (Dinno 2015). This analysis was intended to characterise differences in experimental dosing regimes reported in the literature rather than to infer comparative toxicity among metals. Comparisons of concentrations were restricted to studies involving direct administration via food or body exposure, while environmental exposure studies were excluded because they did not report extractable standardised concentration values; such exposures are typically indirect, heterogeneous, and not controlled. When a study tested multiple concentrations of the same metal, each experimental treatment was recorded as an independent observation in the dataset, allowing the range of dosing regimes reported in the literature to be represented. Metals represented by only a few studies were grouped into an “Other” category, and extreme concentration outliers were removed using the interquartile range (IQR) criterion to minimise the influence of atypical dosing values (Newman and Newman 2014; Walker et al. 2016; Yang et al. 2018; Thériault et al. 2024). Because administered concentrations spanned several orders of magnitude across studies, values were displayed on a log10 scale in the figure to improve graphical clarity; however, all statistical analyses were conducted using the original, untransformed concentration data.

Finally, to test Hypothesis 5 (H5), which concerns the scarcity of studies assessing multi-metal mixtures despite realistic environmental co-exposure scenarios, studies were classified as investigating single-metal or multi-metal exposures. The number of studies in each category was quantified, and differences in frequencies were evaluated using a Chi-square goodness-of-fit test, which assesses whether the observed distribution of study types differs significantly from an expected even allocation. Co-occurrence patterns among metals in multi-metal studies were additionally visualised using a heatmap to identify which metals are commonly tested together and highlight gaps in the literature.

## Results

**Fig. 2:**
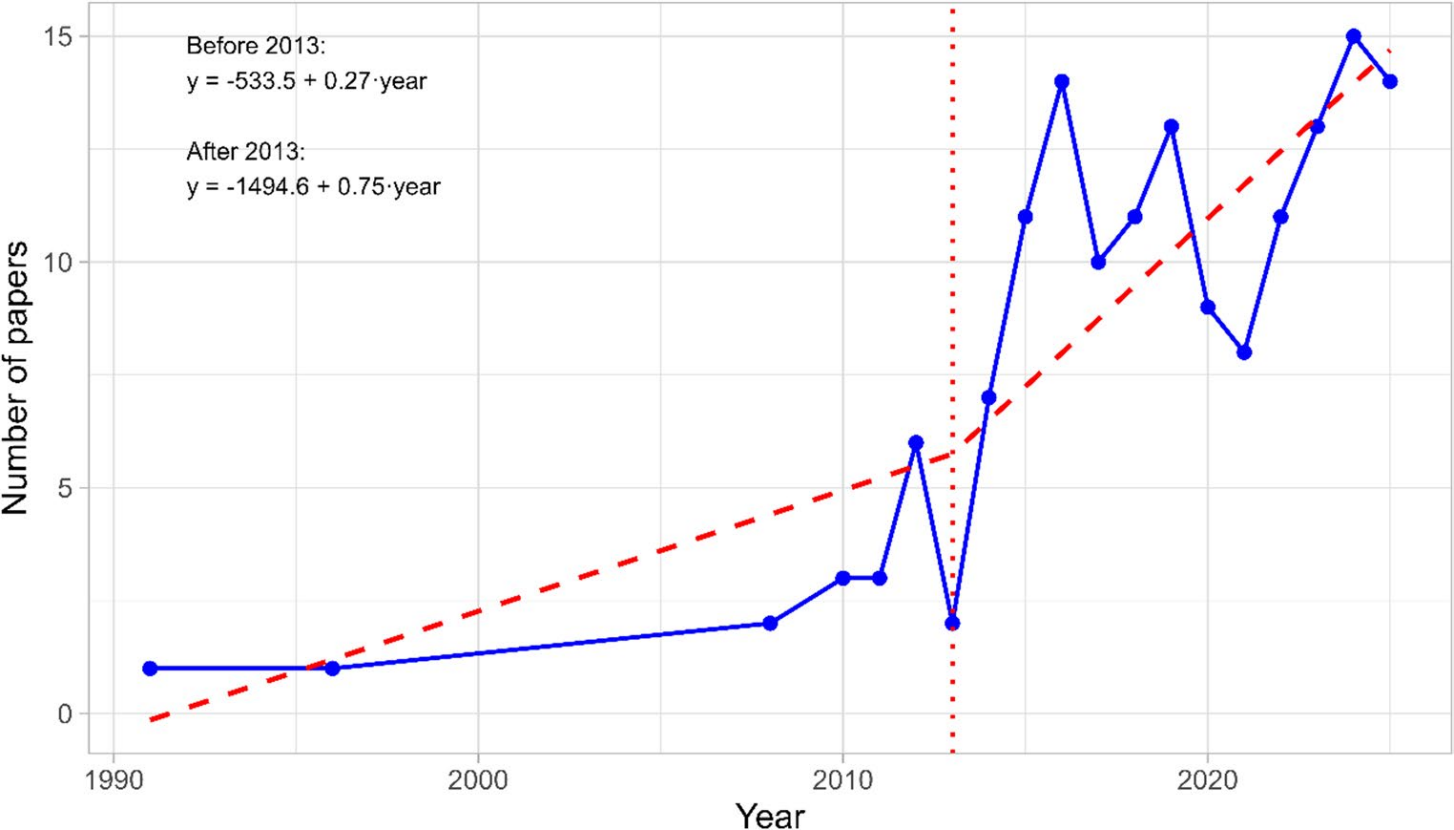
Annual number of articles published on metallic pollutants and bees (1991–2025). A piecewise linear regression was applied to evaluate whether publication trends differed between earlier and more recent periods. The red dashed line shows the fitted model, with a transition point at 2013 (vertical dotted line), yielding separate slopes before and after this year. This approach assesses whether the publication growth rate changed across the two phases, assuming independent residuals and approximately linear trends within each segment. The equations displayed correspond to the fitted regressions for the pre- and post-transition intervals

The number of articles published between 1991 and 2007 remained very low, ranging from zero to two per year. Publication activity increased around 2012, although a brief decline was observed in 2013. Segmented regression identified 2013 as a clear breakpoint, marking the onset of a sustained and significant growth phase. From 2013 onward, research output on metallic pollutants and bees increased steadily and consistently (Fig. 2).

**Fig. 3:**
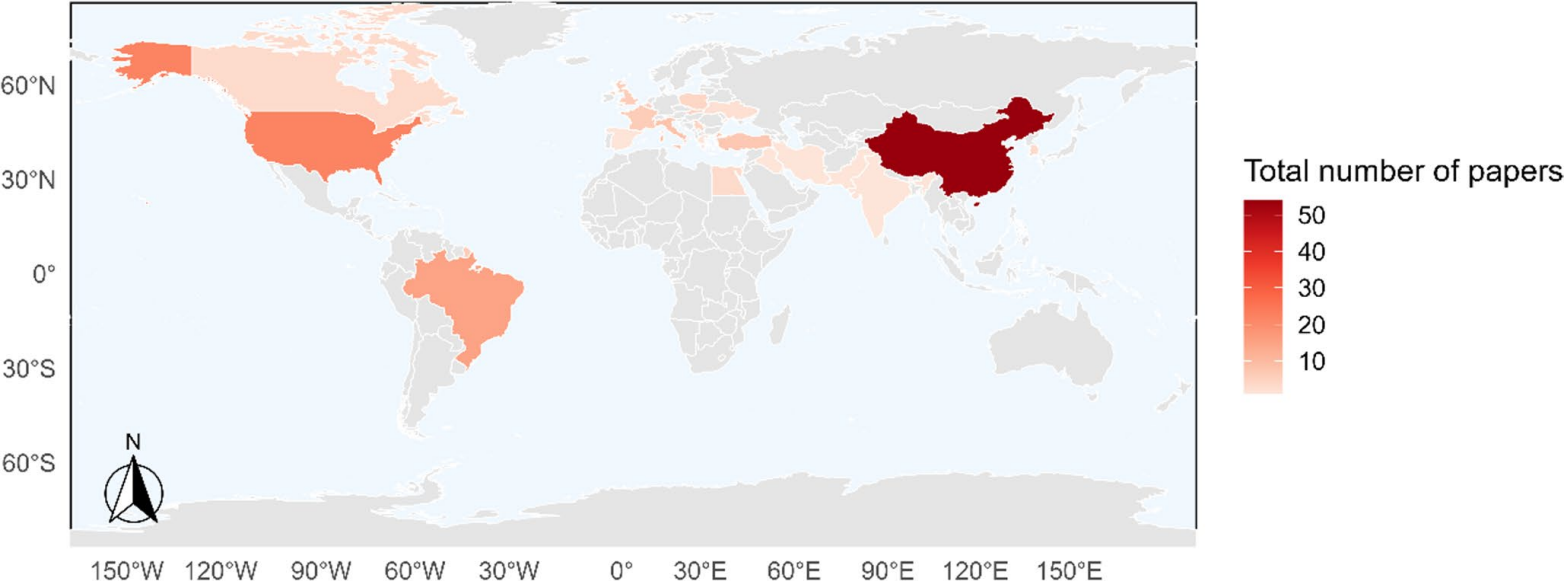
Heatmap illustrating the geographic distribution of studies on the effects of metallic pollutants on bees. Darker colours correspond to a greater number of published articles

Research on the impacts of metallic pollutants on bees is geographically concentrated, with China emerging as the leading contributor, accounting for 54 publications (34.6% of the total dataset) (Fig. 3). The United States ranks second with 22 articles (14.1%), followed by Brazil with 15 studies (9.6%) (Fig. 3). Additional substantial contributions have been made by European and Eurasian countries, including France, the United Kingdom, Turkey, and Italy, highlighting the broad but uneven global distribution of research effort in this field (Fig. 3).

**Fig. 4:**
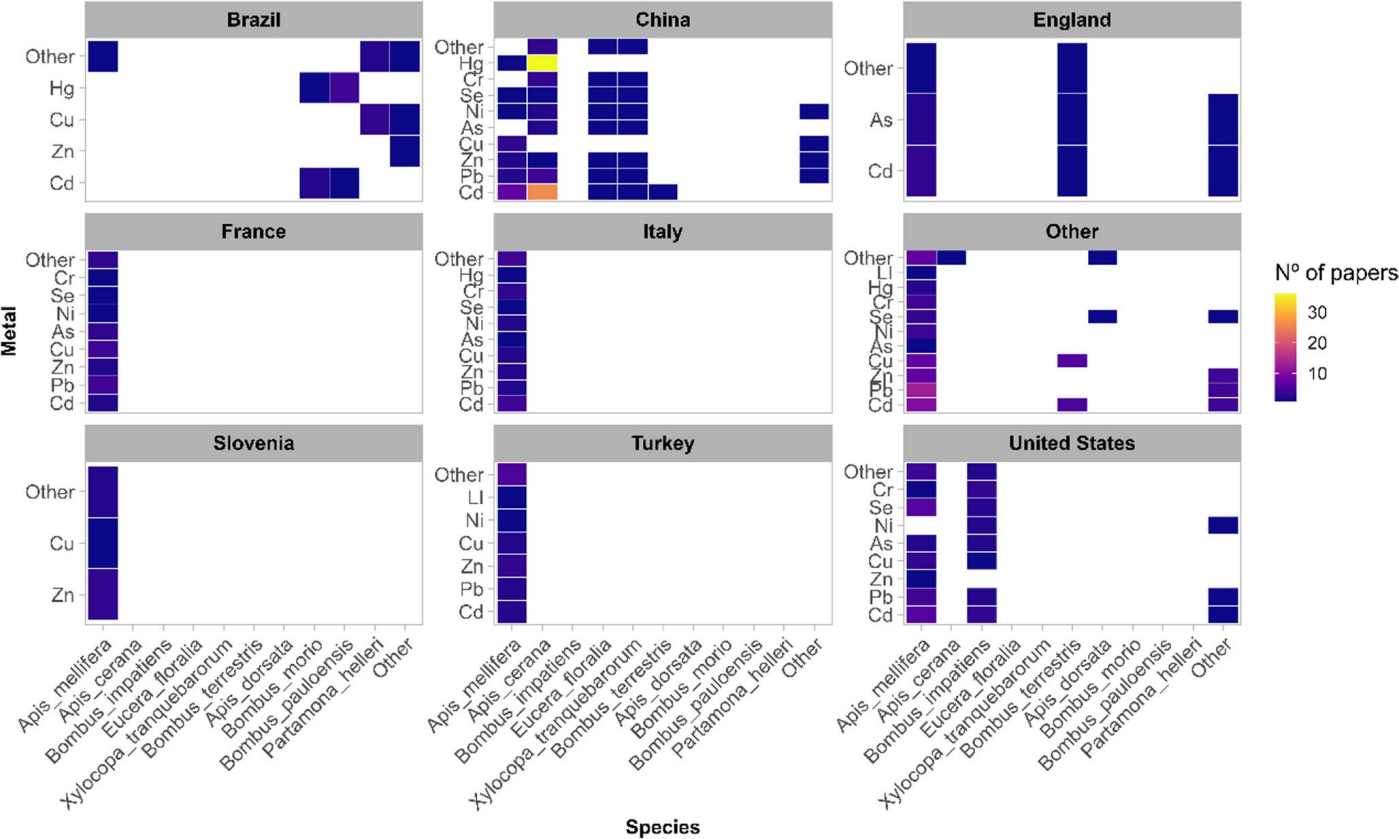
Heatmap showing correlations among countries, bee species, and metals studied in the leading countries. Other: other countries with few studies combined. Metallic pollutants abbreviations: Cd, cadmium; Cu, copper; Hg, mercury; Zn, zinc; Cr, chromium; Se, selenium; Ni, nickel; As, arsenic; Pb, lead; Other, other metals with few studies combined

In Brazil, research has examined a diverse range of bee species, including *(A) mellifera*, *Bombus pauloensis*, *Bombus morio*, and *Partamona helleri* (Fig. 4). Most studies involved *(B) pauloensis* exposed to mercury (*N* = 4, 1.2%) and *Partamona helleri* exposed to copper (*N* = 3, 0.9%), while other combinations were represented by only 1–2 studies (< 1%), indicating that most species–metal combinations remain scarcely investigated. In China, *A. cerana* was the most frequently studied species, particularly in research on mercury (*N* = 36, 10.5%) and cadmium (*N* = 26, 7.6%), whereas other species such as *A. mellifera* and *Eucera floralia* had far fewer studies (generally ≤ 7 studies, < 2%), showing a marked focus on *A. cerana* for key metals. In the United States, studies primarily focused on *A. mellifera* (e.g., selenium: *N* = 6, 1.8%; cadmium: *N* = 4, 1.2%) and *Bombus impatiens* (1–3 studies per metal, 0.3–0.9%), reflecting moderate coverage of multiple metals. By contrast, countries such as France, Italy, Slovenia, and Turkey focused on *A. mellifera*, with metals such as copper, lead, and cadmium investigated by only a small number of studies (1–4, ≤ 1.2%).

The number of studies varied significantly among bee species (H1: χ² = 684.2, df = 18, *p* < 0.001), with *A. mellifera* (70 articles, 48.3%), *A. cerana* (43 articles, 29.7%), and *Bombus terrestris* (9 articles, 6.2%) being the most frequently studied. In contrast, species such as *Pseudapis oxybeloides*, *Xylocopa tranquebarorum*, and *Apis florea* were significantly less studied (1 article, 0.7% each). Adult bees (119 articles, representing 82.1% of the total) were significantly more studied than larvae (8 articles, 5.5%) or multiple life stages within the same study (13 articles, 9%) (χ^2^ = 173.1, df = 2, *p* < 0.001). Workers (136 articles, representing 93.8% of the total) were significantly more studied than queens (1 article, 0.7%), drones (1 article, 0.7%), solitary individuals (4 articles, 2.8%), or studies involving more than one caste (3 articles, 2.1%), which were all markedly underrepresented (χ² = 501.7, df = 4, *p* < 0.001).

Studies differed significantly in the exposure media categories used to assess metal impacts on bees (χ² = 196.2, df = 3, *p* < 0.001). Exposure via food-related media was strongly overrepresented in the literature (112 articles, 73.2%), indicating that most studies relied on dietary administration pathways. In contrast, body-based exposure approaches were significantly underrepresented (10 articles, 6.5%), while environmentally mediated exposures were also less frequent than expected (33 articles, 21.6%).

The 10 most frequently studied metallic pollutants were cadmium (Cd, 50 articles, 34.5%) and mercury (Hg, 23 articles, 15.9%), followed by copper (Cu, 13 articles, 9%), and arsenic (As, 10 articles, 6.9%). The other metals summed 49 studies (33.7% of the total). The number of studies differed significantly among metals (χ² = 386.5, df = 21, *p* < 0.001).

Studies differed significantly in the impact categories used to assess metal effects on bees (χ² = 102.9, df = 6, *p* < 0.001). Physiological endpoints were the most frequently investigated (60 articles, 41.4%) (H2), followed by behavioural and mortality endpoints (24 articles each, 16.6%), and cytology/histology effects (9 articles, 6.2%).

Table 2 summarises the directional Net Effect patterns reported for metallic pollutants across impact classes. Overall, the evidence base was dominated by adverse outcomes, with most metals showing predominantly negative Net Effect scores across multiple biological endpoints. Cadmium (Cd) consistently exhibited strongly negative values across several impact classes, particularly in mortality and physiological responses, indicating a predominance of adverse outcomes in the literature. Similarly, copper (Cu), lead (Pb), and arsenic (As) showed largely negative Net Effect values across behavioural, physiological, and mortality-related endpoints. Even metals with essential biological roles, such as zinc (Zn) and selenium (Se), were more frequently associated with negative than with positive or neutral responses, although occasional neutral or positive outcomes were reported in specific impact classes. Across biological endpoints, physiological and mortality-related responses concentrated the most consistently negative directional evidence, whereas comparatively fewer studies addressed reproductive, colony-level, or microbiome-related impacts.

Because extreme concentrations and long exposure durations could disproportionately influence directional synthesis, we also performed a sensitivity analysis using a Weighted Net Effect index that incorporates both administered concentration and exposure duration. Weighted Net Effect values were generally consistent with the unweighted Net Effect indices, indicating that the overall directional patterns were robust to differences in exposure intensity and duration across studies (Table 2). Differences between the two metrics occurred mainly in cases with balanced positive and negative outcomes, where weighting by exposure intensity altered the relative contribution of individual studies (Table 2). For example, silver in the mortality category shifted from a neutral Net Effect (0) to a positive Weighted Net Effect (1), suggesting that the positive outcome was associated with higher exposure intensity. Similarly, zinc in physiology changed from a neutral Net Effect (0) to a moderately negative Weighted Net Effect (− 0.46), indicating that negative responses were linked to higher concentrations or longer exposures. In contrast, for metals with strongly directional evidence, such as copper in behavioural responses (NE = − 0.9; WNE = − 1), the weighted analysis reinforced the predominance of negative effects.

**Table 2:** Net Effect and Weighted Net Effect values represent the mean directional balance of reported outcomes for each metal–impact combination. Positive effects were coded as + 1, negative effects as − 1, and neutral (no effect) outcomes as 0. The index ranges from − 1 (all reported effects negative) to + 1 (all reported effects positive), with values close to 0 indicating a balance between positive and negative outcomes. For each cell, the Net Effect and Weighted Net Effect values are followed by the number of positive, negative, and neutral records in parentheses. The symbol “–” indicates that the Net Effect or Weighted Net Effect could not be calculated due to the absence of valid observations or because the total weight was equal to zero. Abbreviations: Ag, silver; Al, aluminium; As, arsenic; B, boron; Cd, cadmium; Ce, cerium; Cr, chromium; Cu, copper; Fe, iron; Hg, mercury; K, potassium; Li, lithium; Mg, magnesium; Mn, manganese; Mo, molybdenum; Ni, nickel; Pb, lead; Pt, platinum; Sb, antimony; Se, selenium; Ti, titanium; Zn, zinc. Where

Impact	Metal	Net Effect (*P*/*N*/NT)	Weighted Net Effect (*P*/*N*/NT)
behaviour	Al	-1 (+ 0 / -1 / 0)	-1 (+ 0 / -1 / 0)
behaviour	As	-1 (+ 0 / -4 / 0)	-1 (+ 0 / -4 / 0)
behaviour	B	-1 (+ 0 / -1 / 0)	-1 (+ 0 / -1 / 0)
behaviour	Cd	-1 (+ 0 / -12 / 0)	-1 (+ 0 / -12 / 0)
behaviour	Ce	-1 (+ 0 / -1 / 0)	-1 (+ 0 / -1 / 0)
behaviour	Cu	-0.9 (+ 1 / -19 / 0)	-1 (+ 1 / -19 / 0)
behaviour	Fe	-1 (+ 0 / -2 / 0)	-1 (+ 0 / -2 / 0)
behaviour	K	-1 (+ 0 / -1 / 0)	- (+ 0 / -1 / 0)
behaviour	Li	-1 (+ 0 / -2 / 0)	-1 (+ 0 / -2 / 0)
behaviour	Mg	-1 (+ 0 / -1 / 0)	- (+ 0 / -1 / 0)
behaviour	Mn	-1 (+ 0 / -2 / 0)	-1 (+ 0 / -2 / 0)
behaviour	Mo	-1 (+ 0 / -1 / 0)	-1 (+ 0 / -1 / 0)
behaviour	Ni	-0.67 (+ 1 / -5 / 0)	-1 (+ 1 / -5 / 0)
behaviour	Pb	-0.85 (+ 1 / -12 / 0)	-1 (+ 1 / -12 / 0)
behaviour	Sb	-1 (+ 0 / -1 / 0)	-1 (+ 0 / -1 / 0)
behaviour	Se	-1 (+ 0 / -4 / 0)	-1 (+ 0 / -4 / 0)
behaviour	Ti	-1 (+ 0 / -4 / 0)	-1 (+ 0 / -4 / 0)
behaviour	Zn	-0.75 (+ 1 / -7 / 0)	-1 (+ 1 / -7 / 0)
cytology_histology	B	-1 (+ 0 / -1 / 0)	- (+ 0 / -1 / 0)
cytology_histology	Cd	-1 (+ 0 / -8 / 0)	-1 (+ 0 / -8 / 0)
cytology_histology	Cu	-1 (+ 0 / -2 / 0)	-1 (+ 0 / -2 / 0)
cytology_histology	Hg	-1 (+ 0 / -5 / 0)	-1 (+ 0 / -5 / 0)
cytology_histology	Pb	-1 (+ 0 / -1 / 0)	-1 (+ 0 / -1 / 0)
cytology_histology	Se	1 (+ 1 / -0 / 0)	- (+ 1 / -0 / 0)
cytology_histology	Zn	-1 (+ 0 / -1 / 0)	-1 (+ 0 / -1 / 0)
colony	Ag	1 (+ 1 / -0 / 0)	1 (+ 1 / -0 / 0)
colony	Cd	-1 (+ 0 / -1 / 0)	-1 (+ 0 / -1 / 0)
colony	Pb	-1 (+ 0 / -1 / 0)	- (+ 0 / -1 / 0)
colony	Se	-1 (+ 0 / -1 / 0)	-1 (+ 0 / -1 / 0)
microbiome	Cd	-1 (+ 0 / -3 / 0)	-1 (+ 0 / -3 / 0)
microbiome	Cu	-1 (+ 0 / -2 / 0)	-1 (+ 0 / -2 / 0)
microbiome	Se	-1 (+ 0 / -3 / 0)	-1 (+ 0 / -3 / 0)
microbiome	Ti	-1 (+ 0 / -1 / 0)	-1 (+ 0 / -1 / 0)
mortality	Ag	0 (+ 1 / -1 / 0)	1 (+ 1 / -1 / 0)
mortality	Al	-1 (+ 0 / -1 / 0)	-1 (+ 0 / -1 / 0)
mortality	As	-1 (+ 0 / -9 / 0)	-1 (+ 0 / -9 / 0)
mortality	B	-1 (+ 0 / -2 / 0)	- (+ 0 / -2 / 0)
mortality	Cd	-1 (+ 0 / -55 / 0)	-1 (+ 0 / -55 / 0)
mortality	Cr	-1 (+ 0 / -3 / 0)	-1 (+ 0 / -3 / 0)
mortality	Cu	-1 (+ 0 / -46 / 0)	-1 (+ 0 / -46 / 0)
mortality	Fe	-1 (+ 0 / -3 / 0)	-1 (+ 0 / -3 / 0)
mortality	Hg	-1 (+ 0 / -1 / 0)	-1 (+ 0 / -1 / 0)
mortality	Li	-1 (+ 0 / -5 / 0)	-1 (+ 0 / -5 / 0)
mortality	Mo	1 (+ 3 / -0 / 0)	- (+ 3 / -0 / 0)
mortality	Pb	-1 (+ 0 / -6 / 0)	-1 (+ 0 / -6 / 0)
mortality	Pt	-1 (+ 0 / -2 / 0)	- (+ 0 / -2 / 0)
mortality	Se	-0.67 (+ 1 / -5 / 0)	-1 (+ 1 / -5 / 0)
mortality	Ti	-1 (+ 0 / -4 / 0)	-1 (+ 0 / -4 / 0)
mortality	Zn	-0.67 (+ 1 / -5 / 0)	-1 (+ 1 / -5 / 0)
phenotype	Al	-1 (+ 0 / -1 / 0)	- (+ 0 / -1 / 0)
phenotype	Cd	-1 (+ 0 / -3 / 0)	-1 (+ 0 / -3 / 0)
phenotype	Cu	-1 (+ 0 / -2 / 0)	-1 (+ 0 / -2 / 0)
phenotype	Fe	-1 (+ 0 / -1 / 0)	-1 (+ 0 / -1 / 0)
phenotype	Pb	-1 (+ 0 / -2 / 0)	-1 (+ 0 / -2 / 0)
phenotype	Se	1 (+ 1 / -0 / 0)	- (+ 1 / -0 / 0)
phenotype	Zn	0 (+ 1 / -1 / 0)	-1 (+ 1 / -1 / 0)
physiology	Al	-1 (+ 0 / -1 / 0)	-1 (+ 0 / -1 / 0)
physiology	Cd	-1 (+ 0 / -40 / 0)	-1 (+ 0 / -40 / 0)
physiology	Ce	-1 (+ 0 / -1 / 0)	-1 (+ 0 / -1 / 0)
physiology	Cr	-1 (+ 0 / -1 / 0)	-1 (+ 0 / -1 / 0)
physiology	Cu	-0.67 (+ 1 / -5 / 0)	-1 (+ 1 / -5 / 0)
physiology	Fe	-1 (+ 0 / -2 / 0)	-1 (+ 0 / -2 / 0)
physiology	Hg	-1 (+ 0 / -37 / 0)	-1 (+ 0 / -37 / 0)
physiology	Mn	-1 (+ 0 / -2 / 0)	-1 (+ 0 / -2 / 0)
physiology	Pb	-1 (+ 0 / -8 / 0)	-1 (+ 0 / -8 / 0)
physiology	Se	0 (+ 2 / -2 / 0)	-1 (+ 2 / -2 / 0)
physiology	Ti	-1 (+ 0 / -1 / 0)	-1 (+ 0 / -1 / 0)
physiology	Zn	0 (+ 2 / -2 / 0)	-0.46 (+ 2 / -2 / 0)
reproduction	As	-1 (+ 0 / -1 / 0)	-1 (+ 0 / -1 / 0)
reproduction	Cd	-1 (+ 0 / -4 / 0)	-1 (+ 0 / -4 / 0)
reproduction	Cu	-1 (+ 0 / -2 / 0)	-1 (+ 0 / -2 / 0)
reproduction	Pb	-1 (+ 0 / -1 / 0)	-1 (+ 0 / -1 / 0)
reproduction	Se	-1 (+ 0 / -1 / 0)	-1 (+ 0 / -1 / 0)

**Fig. 5:**
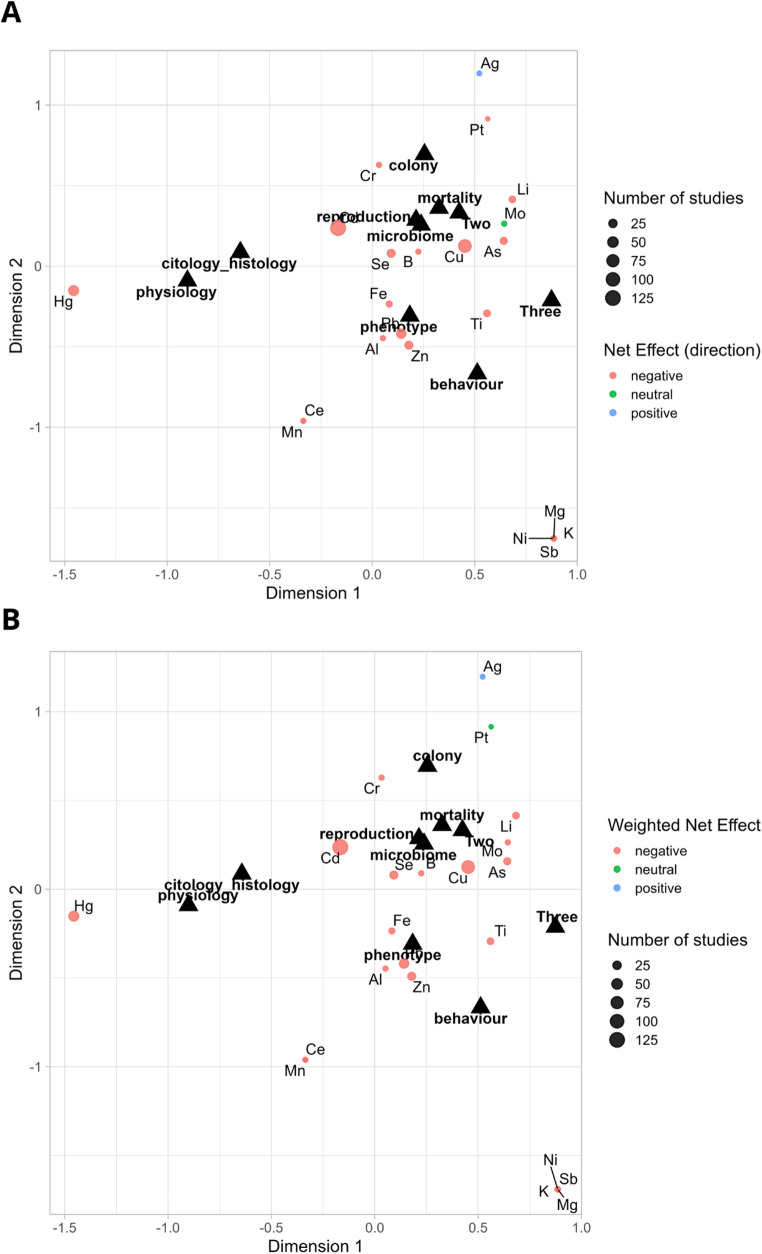
Correspondence Analysis (CA) ordination of metallic pollutants and impact classes based on their co-occurrence frequencies in the literature. Panel A shows the CA map with metals scaled by the number of treatment-level observations and coloured according to the predominant direction of the unweighted Net Effect (positive, negative, or neutral). Panel B presents the same ordination with metals scaled by the absolute magnitude of the weighted Net Effect, incorporating administered concentration and exposure duration. Impact classes are represented as triangles

Correspondence Analysis (CA) provided an exploratory ordination of the thematic structure of the literature by mapping the co-occurrence between metals and impact classes. The overall association between metals and biological endpoints was significant (χ² = 346.13, df = 189, *p* < 0.001), indicating that the distribution of reported effects differed among metal–impact combinations. The first two CA dimensions accounted for 38.16% and 21.39% of the total inertia, respectively. Contributions of individual metals and impact categories to the CA axes indicate that the structure of the first dimension was largely driven by physiological endpoints and highly studied metals such as Hg and Cu, whereas behavioural and mortality endpoints contributed more strongly to the second dimension. When Net Effect scores were overlaid onto the ordination, the most intensively investigated associations were generally characterised by predominantly negative directional evidence (Fig. 5). Detailed CA statistics, including eigenvalues and variable contributions to each dimension, are provided in Supplementary Material 1.

To evaluate whether essential metals differed systematically from non-essential toxic metals in the predominant direction of reported outcomes (H3), metals were grouped by biological role and compared using Net Effect scores. Results indicated that essential metals (Zn, Cu, Se) had significantly less negative Net Effect values compared with non-essential toxic metals (Cd, Pb, Hg) (mean NE essential = − 0.62 vs. non-essential − 0.99; Wilcoxon rank-sum test, W = 393, *p* = 0.0007), suggesting a relatively milder impact of essential metals on reported outcomes. However, when the Weighted Net Effect index, which incorporates both administered concentration and exposure duration, was considered, differences between essential and non-essential metals were no longer statistically significant (mean WNE essential = − 0.97 vs. non-essential − 1; Wilcoxon rank-sum test, W = 252, *p* = 0.294). These findings indicate that the overall directional patterns of effects were robust to differences in exposure intensity and duration, and that apparent differences in unweighted Net Effect were largely driven by treatments with lower concentrations or shorter exposure times.

**Fig. 6:**
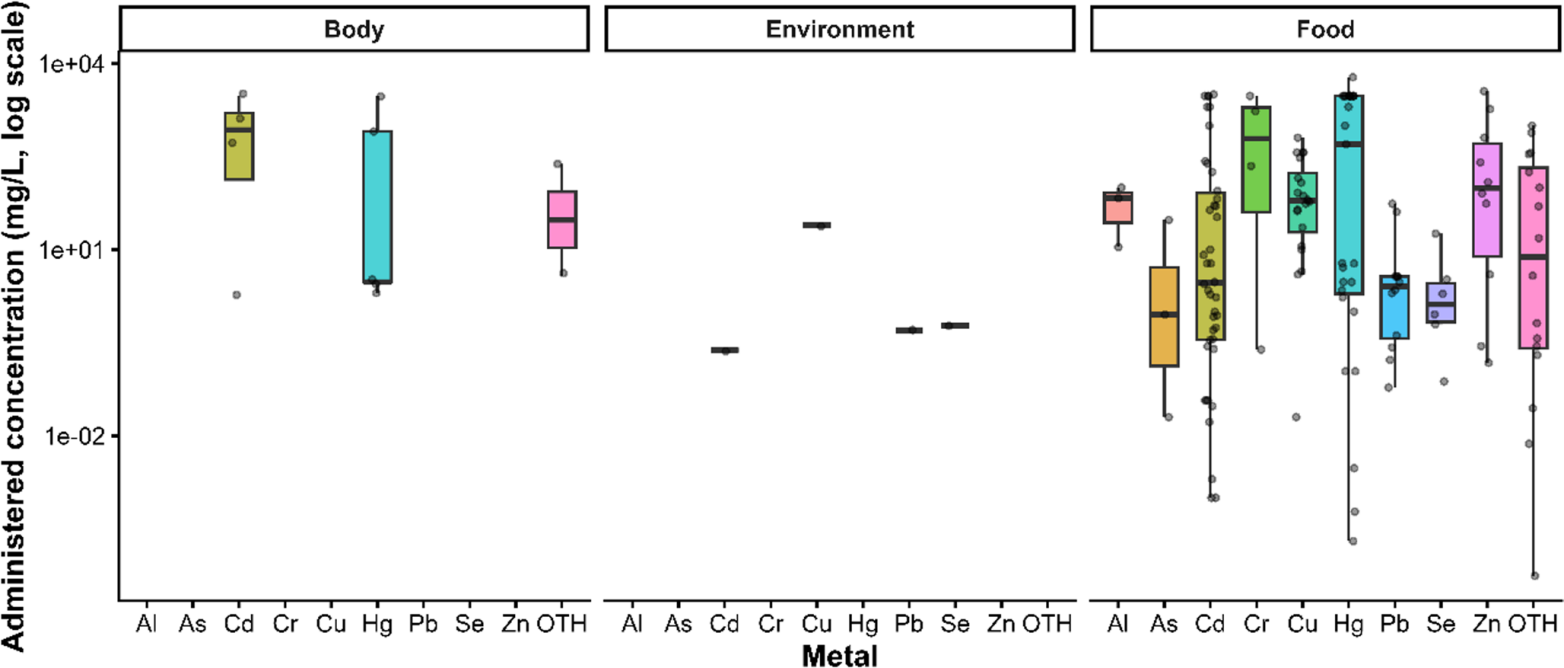
Administered concentrations of metallic pollutants in experimental studies evaluating their effects on bees (y-axis shown on a log10 scale) for Body, Environment and Food exposure media. Abbreviations: Al, aluminium; As, arsenic; Cd, cadmium; Cr, chromium; Cu, copper; Hg, mercury; Pb, lead; Se, selenium; Zn, zinc; OTH, metals with low study frequency grouped into a single category

Administered experimental concentrations differed among metals when studies using food-based exposure were considered (Kruskal–Wallis: χ² = 19.34, df = 9, *p* = 0.022) (Fig. 6). However, post-hoc Dunn’s tests with Bonferroni correction did not detect any statistically significant pairwise differences among metals, indicating that the overall heterogeneity was not driven by specific metal pairs. In contrast, no significant differences in administered concentrations among metals were observed for body exposure routes (χ² = 0.58, df = 2, *p* = 0.748) or environmental exposure routes (χ² = 3.00, df = 3, *p* = 0.392) (Fig. 6).

**Fig. 7:**
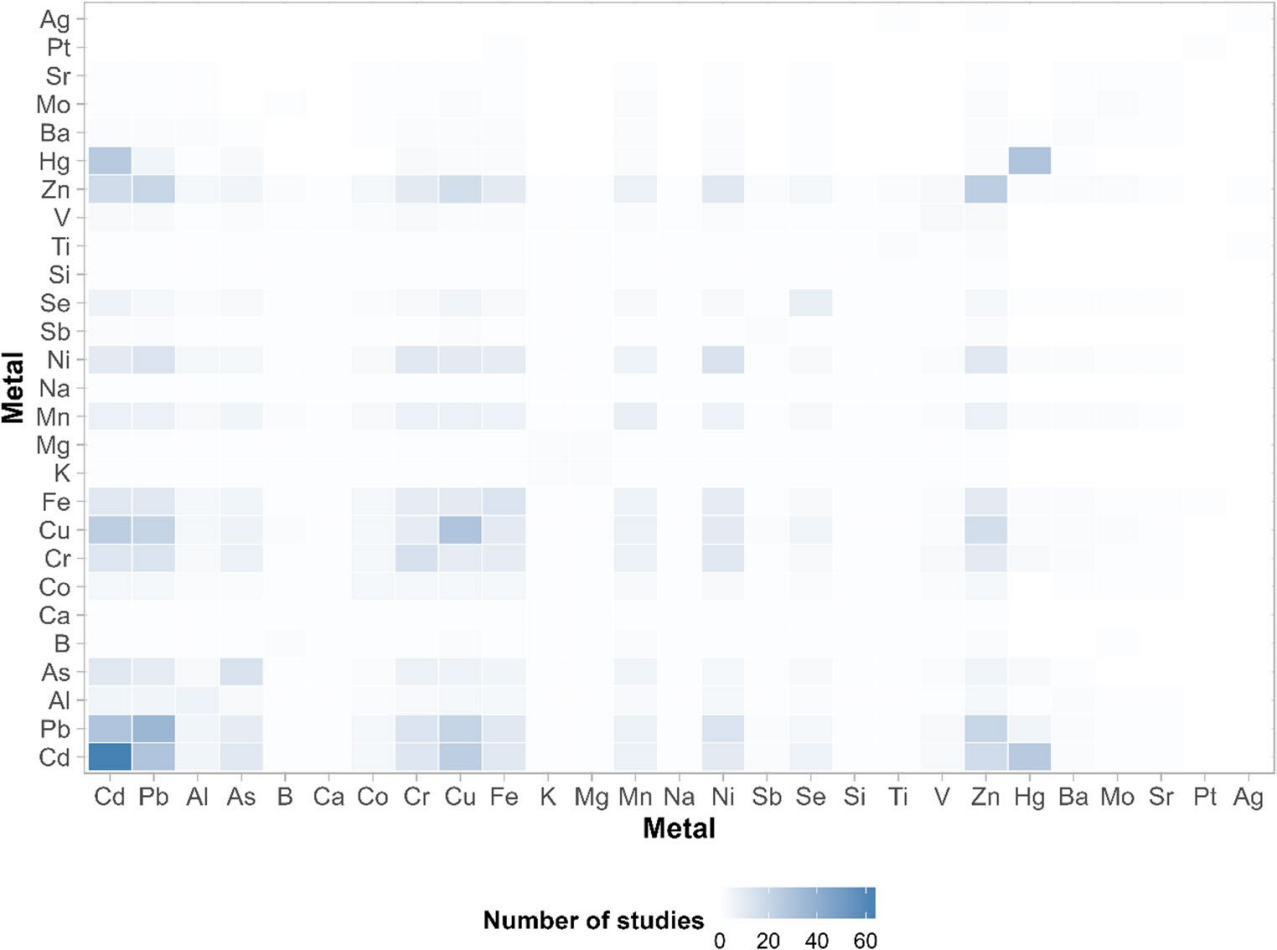
Heatmap showing the co-occurrence of metals in studies assessing multi-metal exposures. Cell colour intensity represents the number of studies in which each pair of metals was tested together. Darker shades indicate higher co-occurrence, while lighter shades indicate less frequent combinations

To test Hypothesis 5 (H5), studies were classified as single-metal or multi-metal exposure. Of the total dataset, 73 studies assessed multi-metal exposures, whereas 59 focused on a single metal. A Chi-square goodness-of-fit test indicated that the difference in study counts between single- and multi-metal designs was not statistically significant (χ² = 0.32, df = 1, *p* = 0.57), suggesting that multi-metal studies are not disproportionately rare in the reviewed literature. Co-occurrence patterns among metals in multi-metal studies were visualised in a heatmap (Fig. 7), highlighting which metals are most frequently tested together and revealing gaps in multi-metal research combinations.

## Discussion

The findings of this study reveal consistent patterns of research concentration and important blind spots in the global literature on metallic pollution and bees. Overall, our hypotheses were partially corroborated. Research effort was disproportionately focused on a few model species, adult life stages and castes, and dietary exposure pathways (supporting H1). Impact endpoints were strongly skewed toward physiological, behavioural, and mortality-related outcomes (partially supporting H2). Net Effect synthesis indicated that essential metals exhibited less negative directional outcomes than non-essential metals, although this difference disappeared when exposure intensity and duration were incorporated using the Weighted Net Effect index (partially supporting H3). Experimental concentrations showed overall heterogeneity among metals, but no significant pairwise differences after post hoc correction (providing only partial support for H4). Finally, contrary to expectations, multi-metal studies were not statistically underrepresented (rejecting H5), although important gaps remain in the combinations of metals investigated.

Publications addressing metallic pollutants and bees have increased steadily since 2013, likely reflecting growing concern about global pollinator declines and the intensification of environmental monitoring in agricultural and industrial landscapes (Duzgoren-Aydin et al. 2006; Porrini et al. 2016; Barbosa et al. 2017; Cai et al. 2019; Jamal et al. 2019; Shi et al. 2019; Demeneix 2020; Papa et al. 2021). This trend highlights the growing recognition of metal contamination as an emerging stressor that interacts with other drivers of bee decline.

Geographic patterns in research were uneven, with China, the United States, and Brazil emerging as leading contributors. This distribution may reflect both the economic relevance of apiculture and these countries’ dependence on pollination services for agricultural productivity (European Union 2023; FAO 2024). Another contributing factor may be the growing dependence on pollination services to sustain agricultural productivity amid declining bee populations and mass colony losses reported worldwide (Al Naggar et al. 2018). From 2000 to 2020, China, the United States, and Brazil ranked among the top three global grain producers (FAO 2024), underscoring their reliance on pollination. However, this geographic pattern is not unique to studies on metallic pollutants. Analyses of the broader scientific literature on bees and pollinators indicate that research output is often concentrated in countries with strong scientific infrastructure, high research investment, and large agricultural sectors, which tend to dominate global scientific production (Vit et al. 2025; Dorey et al. 2026). Nevertheless, the concentration of studies in a limited number of countries also suggests that large regions with substantial metal pollution problems remain underrepresented, potentially constraining global ecological risk assessments.

Consistent with Hypothesis 1, research has relied heavily on a small number of managed model species, particularly *A. mellifera* and *A. cerana*. This taxonomic bias is unsurprising given their economic importance, their wide geographic distribution, and ease of laboratory handling (Han et al. 2012; Arena and Sgolastra 2014; Sanchez-Bayo and Goka 2014). However, such dependence limits ecological generalisation, as wild bees differ markedly in nesting biology, foraging behaviour, and exposure routes. Furthermore, adult workers predominated in experimental designs, whereas larval stages and multi-stage approaches were rare. The collection and handling of adult bees are generally easier, and sublethal effects can be more readily observed at this stage (European Food Safety Authority 2013). In contrast, experiments with larvae require a specialised diet and more stringent laboratory conditions than those for adult bees (Di et al. 2016). Toxicity assessment, for example, showed that lethal concentrations of copper for adults were found approximately ten times higher than for larvae, while cadmium lethal concentrations for adults were about 200 times higher than for larvae (Di et al. 2020). These findings indicate that studies focusing solely on adult bees may underestimate the actual impacts of metallic pollutants on the colony. Consequently, evaluating the effects of metals on larval stages is essential to fully understand the ecological risks posed to these pollinators. Exposure media were also strongly skewed toward food-based administration, with body-based and environmentally mediated pathways significantly underrepresented. These methodological patterns indicate that the current evidence base may not adequately capture the diversity of realistic exposure scenarios experienced by wild pollinators.

Hypothesis 2 was almost fully corroborated. Impact endpoints were unevenly distributed across the literature, with physiological responses accounting for the largest share of studies, followed by behavioural and mortality-related outcomes. Colony-level, reproductive, and microbiome impacts remained particularly underrepresented. This imbalance confirmed that research attention is disproportionately allocated across impact types. Such bias likely reflects the methodological convenience of measuring acute physiological endpoints, whereas colony development, reproduction, and microbiome-mediated processes require longer-term and more complex experimental frameworks (Gekière et al. 2023; Li et al. 2024; Frizzera et al. 2024; Esmaeily et al. 2025). This gap is particularly important because many environmental stressors act primarily through sublethal mechanisms that alter key biological processes across the bee life cycle, including mating behaviour, nesting success, reproductive output, and juvenile development (Gekière et al. 2025). Such disruptions may reduce individual fitness and, when accumulated across generations, ultimately translate into population-level declines even in the absence of strong acute mortality signals. Consequently, studies focusing primarily on short-term physiological responses may overlook ecologically relevant pathways through which metallic pollutants affect pollinator populations.

Correspondence analysis further demonstrated that the thematic structure of the literature clusters around a restricted set of frequently studied metal–endpoint combinations, particularly involving physiological endpoints and highly studied metals such as mercury and copper. Mercury, for instance, has been shown to alter gene and brain protein expression, disrupt enzyme activity, and induce DNA damage, ultimately leading to cytological injury, apoptosis, and mortality (Zhang et al. 2014; Liu et al. 2016; Nogueira et al. 2019; Bai et al. 2023; Balsamo et al. 2023). Copper exposure has been linked to behavioural impairments in walking, foraging, feeding, and learning, as well as to mortality across life stages and to disruptions in the gut microbiota and reproduction (Hladun et al. 2015b; Rothman et al. 2020; Bernardes et al. 2022; Botina et al. 2023). Net Effect overlays further indicated that these dominant research areas are characterised predominantly by negative directional evidence, consistent with the ecotoxicological focus on detecting detrimental responses to contaminants. Although adverse outcomes clearly dominate the evidence base, responses were not entirely uniform. A small number of metal–endpoint combinations showed positive directional balances, including selenium in cytology/histology and phenotype endpoints (NE = 1), silver at the colony level (NE = 1), and molybdenum in mortality responses (NE = 1), while other cases, such as selenium and zinc in physiological endpoints, exhibited balanced responses (NE ≈ 0). These isolated cases likely reflect context-dependent responses arising under specific experimental conditions, rather than genuine beneficial effects. Overall, this pattern suggests that the current knowledge base is shaped both by uneven research effort and by a strong concentration of studies on adverse outcomes within a limited set of experimental contexts, leaving other exposure scenarios and biological responses comparatively underexplored.

Hypothesis 3 was partially supported. Essential metals such as Zn, Cu, and Se exhibited significantly less negative Net Effect values than non-essential toxic metals such as Cd, Pb, and Hg. However, this difference disappeared when exposure intensity and duration were incorporated using the Weighted Net Effect index, indicating that the apparent mitigation associated with essential metals was largely driven by treatments with lower exposure intensity or shorter experimental duration rather than by intrinsic differences in toxicity. Experimental concentrations also varied among metals in food-based exposure studies, although post-hoc comparisons did not reveal specific pairwise differences, suggesting that heterogeneity in administered doses contributes to the observed patterns across studies. This suggests that, despite their physiological roles, essential metals are typically investigated under exposure conditions that still elicit harmful effects, underscoring that essentiality does not imply safety when concentrations exceed biological thresholds (Rengel 2004; Tchounwou et al. 2012; Monchanin et al. 2021c; Naccari et al. 2025). Such patterns are consistent with the well-established principle that essential trace elements operate within a narrow window between nutritional requirement and toxicity, beyond which homeostatic regulation is disrupted, and adverse physiological effects emerge (Fraga 2005; Maret 2016).

Indeed, even metals that may exert beneficial effects at trace levels, such as zinc and selenium, frequently produce adverse behavioural, physiological, and mortality-related outcomes when exposure increases (Milivojević et al. 2015; Zhang et al. 2015; Rothman et al. 2019; Chi et al. 2020). Additional pollutants such as arsenic, aluminium, and chromium further highlight the breadth of metallic stressors affecting bees, with evidence for increased mortality, impaired learning, lipid peroxidation, and gene deregulation (Gauthier et al. 2016; Hesketh et al. 2016; Heard et al. 2017; Sgolastra et al. 2018; Bai et al. 2023). Taken together, these findings indicate that the overall negative directional signal detected across both essential and non-essential metals reflects the predominance of toxic responses at experimentally relevant exposure levels, rather than fundamental differences in the biological nature of these elements.

Hypothesis 4 received only partial support. Although the overall Kruskal–Wallis test indicated heterogeneity in administered concentrations among the metals, post hoc comparisons did not detect statistically significant pairwise differences. This heterogeneity complicates cross-metal comparisons and limits ecological realism, as experimental dosing regimes often span orders of magnitude beyond environmentally relevant exposure levels (Chapman 2002; Martin et al. 2021). Ideally, experimental concentrations should be compared with field-measured concentrations to determine whether exposure scenarios are environmentally realistic. However, such comparisons were not feasible within our dataset because most studies reported metal concentrations in bee-related matrices (e.g., bee bodies, honey, or pollen) rather than in environmental compartments such as soil, nectar, or water, and reported units and exposure metrics were highly heterogeneous across studies. Because no pairwise differences were statistically significant after correction for multiple comparisons, the observed heterogeneity likely reflects broad variability in experimental designs rather than systematic differences among specific metals. This limitation prevents a robust classification of experimental treatments as field-realistic or unrealistic, highlighting an important gap in the current literature. Such discrepancies only partially reflect established regulatory thresholds for metals in water (Briffa et al. 2020) and are concerning given that negative effects on bees have been reported even at concentrations near or below permitted limits (Abdalla and Domingues 2015; Abdalla et al. 2018; Nogueira et al. 2019; Ceschi-Bertoli et al. 2020; Boeing et al. 2024). Greater standardisation and stronger alignment with field-realistic concentrations are needed to improve comparability and the relevance of risk assessments.

Finally, Hypothesis 5 was not corroborated. Multi-metal studies were not significantly less common than single-metal designs. Nonetheless, the co-occurrence heatmap revealed that only a limited subset of metal combinations has been repeatedly tested. Thus, while multi-metal approaches are present, the literature still falls short of capturing the complexity of realistic environmental co-exposure mixtures (Monchanin et al. 2023). This gap is notable given that bees are naturally exposed to multiple metallic pollutants simultaneously. Moreover, combined exposures may generate antagonistic, additive, or synergistic interactions, making their ecological consequences difficult to predict from single-metal evidence alone (Di et al. 2020; Monchanin et al. 2021a, c). Future work should extend beyond pairwise combinations and incorporate interactions with other major stressors, such as pesticides, pathogens, and nutritional limitation (Goulson et al. 2015; Cunningham et al. 2022).

In conclusion, this scientometric synthesis demonstrates that knowledge production on metallic pollution and bees is structured by strong taxonomic, methodological, and thematic biases. The field remains dominated by studies on *A. mellifera*, adult workers, dietary exposure routes, and acute physiological endpoints, while colony-level, reproductive, microbiome, and wild bee impacts remain major blind spots. Addressing these gaps will require broader taxonomic coverage, more ecologically realistic exposure designs, greater attention to chronic and sublethal endpoints, and integration of complex multi-stressor scenarios. Such advances are essential for developing robust and generalisable risk assessments of metallic contamination in pollinator communities. Finally, it is important to note that this synthesis was based on literature indexed in the Web of Science, and relevant studies available in other databases or grey literature may not have been captured. Future reviews could therefore expand database coverage to provide an even more comprehensive representation of research on metallic pollutants and bees.

## Supplementary Information

Below is the link to the electronic supplementary material.

Supplementary Material 1

## Data Availability

Martins, Fernando; Azevedo, Cristiano; Rocha, Diego; Melo, Diego; Ribeiro, Marcelo; Moura, Sandra (2026), “Biases and blind spots in the global research agenda on metallic pollution and bees”, Mendeley Data, V1, doi: 10.17632/m4w4vyjf73.1.
